# Intramyocardial Injection of Recombinant Adeno-Associated Viral Vector Coexpressing PR39/Adrenomedullin Enhances Angiogenesis and Reduces Apoptosis in a Rat Myocardial Infarction Model

**DOI:** 10.1155/2017/1271670

**Published:** 2017-03-02

**Authors:** Rui An, Cong Xi, Jian Xu, Ying Liu, Shumiao Zhang, Yuemin Wang, Yuewen Hao, Lijun Sun

**Affiliations:** ^1^Department of Radiology, Xijing Hospital, Fourth Military Medical University, Xi'an, China; ^2^Department of Neurology, Baoji City People's Hospital, Baoji, China; ^3^Department of Physiology, Fourth Military Medical University, Xi'an, China

## Abstract

Cotransfer of angiogenic and antiapoptotic genes could be the basis of new gene therapy strategies for myocardial infarction. In this study, rAAV-PR39-ADM, coexpressing antimicrobial peptide (PR39) and adrenomedullin (ADM), was designed with the mediation of recombinant adeno-associated virus. In vitro, CRL-1730 cells were divided into four groups, namely, the sham group, the AAV-null group, the NS (normal saline) group, and the PR39-ADM group. Immunocytochemistry analysis, CCK-8 assays, Matrigel assays, and apoptotic analysis were performed; in vivo, myocardial infarction model was established through ligation of the left coronary artery on rats, and treatment groups corresponded to those used in vitro. Myocardial injury, cardiac performance, and the extent of myocardial apoptosis were assessed. Results suggested that rAAV-PR39-ADM administration after myocardial infarction improved cell viability and cardiac function, attenuated apoptosis and myocardial injury, and promoted angiogenesis. Subsequently, levels of 6×His, HIF-1*α*, VEGF, p-Akt, Akt, ADM, Bcl-2, and Bax were measured by western blot. rAAV-PR39-ADM increased p-Akt, HIF-1*α*, and VEGF levels and induced higher Bcl-2 expression and lower Bax expression. In conclusion, our results demonstrate that rAAV-PR39-ADM mitigates myocardial injury by promoting angiogenesis and reducing apoptosis. This study suggests a potential novel gene therapy-based method that could be used clinically for myocardial infarction.

## 1. Introduction

Coronary heart disease, with myocardial infarction (MI) as the acute manifestation, remains the leading threat to human health worldwide [1–4]. Numerous treatment methods based on the pathogenesis and related to timely reperfusion [5, 6] and reinfarction or ventricular remodelling reduction in the later stage of MI using pharmacological agents have flourished recently [7].

The therapeutic delivery of nucleic acid polymers into cells, as a drug to treat disease, is known as gene therapy; this approach has many components including adrenergic manipulation, Ca cycling protein handling, angiogenesis, cardiac regeneration, and targeting cardiac arrhythmias [8]. In general, antiapoptosis, proangiogenesis, and the dilation of corresponding vessels are the main aspects of MI treatment. Inhibiting apoptosis in both cardiomyocytes and endotheliocytes might reduce the likelihood of reinfarction at the intracellular level. Collateral vessel growth induced by growth factors such as vascular endothelial growth factor (VEGF) and the dilation of corresponding vessels induced by vasodilation substances such as prostaglandin and adrenomedullin supply the at-risk myocardium with necessary oxygen and nutrition and deplete the tissue of metabolites. However, previous gene therapy approaches have usually focused on the role of a single protective gene on cytoprotection, vessel dilation, or angiogenesis of the myocardial infarction, and the therapeutic effects were actually modest.

PR39, a proline- (P-)/arginine- (R-) rich antimicrobial peptide of 39 amino acids, was originally isolated from pig intestine and identified in neutrophil azurophilic granules and macrophages [9, 10]. Previous studies have demonstrated that PR39 plays an important role in reducing cardiac injury through the induction of angiogenesis by inhibiting HIF-1*α* degradation [11] and in antiapoptosis through the expression of IAP-2 [12].

Adrenomedullin (ADM), a potent and long-lasting vasoactive peptide, was originally isolated from human pheochromocytoma in 1993 [13]. Its functions including dilating the coronary artery, inhibiting vascular remodelling, and reducing fibroblast proliferation and extracellular matrix synthesis result in therapeutic effects such as reducing infarct size, ischemia-reperfusion injury, and ischemia-induced arrhythmias. This consequently lowers mortality in rodent models, according to previous reports [14–19].

Accordingly, based on previous work [20, 21], an adeno-associated viral vector coexpressing PR39 and ADM was constructed. The aim of this study was to assess this strategy for the protection of the jeopardized myocardium through both cytoprotection and angiogenesis (vectors were constructed with the support of Xi'an HuaGuang Bioengineering Co. (Xi'an, China) and China patents were applied (CN1919344A, CN1919343A, CN100589846C, and CN100589847C)).

## 2. Materials and Methods

### 2.1. Cell Culture

Immortalized CRL-1730 cells and HEK-293 cells (provided by Xi'an HuaGuang Bioengineering Corporation, Shaanxi Province of China) were maintained in RPMI 1640 complete medium (Sigma Corporation, USA) with supplemental growth factors and antibiotics according to company specifications. Hypoxia condition was performed with 1% O_2_ and 5% CO_2_ cell incubator (provided by the Department of Physiology of the Fourth Military Medical University, Xi'an, China).

### 2.2. Strains and Plasmids

Bacillus coli TOP10, T/TAT-His and pBV220/NT4, AAV vector pSSHG/CMV that contains 3′LTR and 5′LTR, and two helper plasmids (pAAV/Ad, PFG140) were provided by Xi'an HuaGuang Bioengineering Corporation, Shaanxi Province of China. pGEM-T-Easy was supplied by Promega Corporation, USA. T4 DNA ligase was supplied by Fermentas Corporation, USA.* Nae *I,* Bam*H I,* Eco*R I,* Eco*721, and Taq DNA polymerase were bought from Xi'an Hua Mei Bioengineering Corporation, Shaanxi Province of China.

### 2.3. Construction of NT4-Intron-6×His-PR39-Splicing-ADM Box

Recombinant propeptide was used to realize bioactive peptide mediated gene therapy (China Patent Numbers: CN1919344A, CN1919343A, and CN100589846C). Recombinant vector (HRE-NT4-Intron-6×His-PR39-splicing-ADM box) was constructed by the connection of cDNA of PR39, ADM, Intron and signal peptide (NT4), hypoxia-responsive element (HRE), and splicing. Then, vectors were transduced into target cells with the help of plasmid (China Patent Number CN100589847C); following transcription of pre-mRNA, two kinds of biological active peptides (PR39 and ADM) were expressed. We adopted the technology of multiple proteins and peptides secretory expression with the guidance of single signal peptide and realized the expression of PR39 and ADM in the same carrier. Vectors were testified by Sangon Biotech (Shanghai) Co., Ltd.

### 2.4. Immunocytochemistry Analysis

First, CRL-1730 cells were cultured for 12 hours in RPMI 1640 medium with 10% fetal bovine serum and were, respectively, transfected with 100 *μ*L 3.4 × 10^9^ p.f.u. AAV-NT4-TAT-6His-PR39-ADM (abbreviated as PR39-ADM below) or equal volume of AAV-null for 48 hours [NS (normal saline) was performed as control]. Cells were washed twice with PBS, fixed with acetone for 15 min, and soaked in 0.75% H_2_O_2_ for 30 min and 0.5% Triton X 100 for 30 min. Then cells were incubated for 4 hours with antibodies against 6×His (1 : 500) and enzyme-labeling antibodies against goat IgG (1 : 500), for DAB coloration. The expression of the fusion proteins was indirectly verified by 6×His expression in cells.

Second, CRL-1730 cells were pretransfected with PR39-ADM or AAV-null for 48 hours (NS was performed as control) and then incubated in hypoxia (37°C, 1% O_2_, N_2_, and 5% CO_2_) for 10 hours; the expression of HIF-1*α* was detected with the incubation of its antibody (1 : 500, method mentioned above).

Digital photomicrographs of the 6×His-positive—and HIF-1*α*-positive—cells were taken from five randomly chosen fields per section to estimate the intensity of protein 6×His and HIF-1*α* expression, which were described as optical density (OD) value by the Image Pro Plus (IPP) image processing software.

### 2.5. CCK-8 Assay

Cell proliferation was detected by Cell Counting Kit-8 (CCK-8) (Sigma-Aldrich, St. Louis, MO, USA) assays. The assays were performed at 12 hours, 24 hours, and 36 hours with different titers of PR39-ADM (data not shown); the effect of hypoxia on CRL1730 cells was also tested. AAV-null and NS were established as control groups. All CRL-1730 cells were incubated with 10 *μ*L of CCK-8 tetrazolium salt for 2 hours, and the absorbance was detected by microplate spectrofluorometer at a 450 nm wavelength. The proliferation experiments were repeated three times with triplicate wells for each condition.

### 2.6. Matrigel Assay

Prior to Matrigel (BD Bioscience, Bedford, MA, USA) assays, the CRL-1730 cells were pretreated with PR39-ADM, AAV-null, and NS for 48 hours. 96-well cell culture plate was coated with 50 *μ*L of ice cold Matrigel as a tube formation base. After allowing the gel to settle for 30 min in a 37°C, 5% CO_2_ incubator, the endothelial cells (5 × 10^4^ per well) from different groups were seeded onto the Matrigel and incubated at 37°C in a 5% CO_2_ incubator. 12 hours after incubation, the extent of tube formation was then recorded using microscope [22].

### 2.7. Apoptotic Analysis

CRL-1730 cells were pretransfected with 100 *μ*L 3.4 × 10^9^ p.f.u. PR39-ADM or equal volume of AAV-null (NS was performed as control) in 37°C, 5% CO_2_ incubator for 48 hours. Then, three groups of cells were placed in the hypoxic incubator (37°C, 1% O_2_, N_2_, and 5% CO_2_) for 10 hours, digested by trypsin, and centrifugated and fixed by absolute alcohol. All groups' apoptosis rates were determined by flow cytometry (FCM).

### 2.8. Animals

All experiments were performed on healthy adult male Sprague-Dawley rats (body weight: 220 g–250 g) that were obtained from the Fourth Military Medical University Animal Center. The rats were kept under pathogen-free conditions at about 22°C on a 12-hour light-dark cycle with free access to food and water. The present study was performed in accordance with the Guide for the Care and Use of Laboratory Animals, published by the US National Institutes of Health (National Institutes of Health Publication Number 85-23, revised 1996) and the experimental protocol was approved by the Ethics Committee of the Fourth Military Medical University.

### 2.9. Production of Acute Myocardial Infarction and Intramyocardial Gene Transfer

Rats were anaesthetized with 3.5% chloral hydrate by intraperitoneal injection. Next, rats were ventilated by respiratory mask (independently developed by the Department of Physiology of the Fourth Military Medical University, Patent Number: ZL200810150927.8) with a pressure-controlled ventilator. After thoracotomy, MI was induced by permanent ligation of the left anterior descending coronary artery (LAD) with a 7-0 polypropylene suture. Subsequently, chest and skin were closed in layers. sham-operated animals were subjected to the same procedures as the experimental animals just without the LAD ligation. PR39-ADM, AAV-null, or NS was, respectively, injected directly into the ischemic border zone of the myocardium by using an insulin syringe with a 30-gauge needle at five separate sites (60 *μ*L to each site). The chest was closed and the animals were allowed to recover immediately after injection.

### 2.10. Experimental Protocol

Fifty-six rats were randomly assigned to four groups: the sham group (*n* = 14) received the sham operation and no LAD ligation; MI groups including the MI + PR39-ADM group (*n* = 14) received the LAD ligation and PR39-ADM (300 *μ*L, 3 × 10^9^ p.f.u.); the MI + AAV-null group (*n* = 14) received the LAD ligation and the same quantity of AAV-null; the MI + NS group (*n* = 14) received the LAD ligation and the same quantity of normal saline.

### 2.11. Evaluation of Cardiac Function by Echocardiography and Invasive Hemodynamic Assessment

Transthoracic echocardiographic examinations were established under isoflurane anesthesia (2%) of rats in each group 4 weeks after MI. An ACUSON echocardiography instrument equipped with a 13 MHz phased-array transducer (Siemens, USA) was used to obtain echocardiographic images. The M-mode images of left ventricular (LV) dimensions were obtained. The left ventricular ejection fraction (LVEF) and left ventricular fractional shortening (LVFS) were recorded. All the above measurements represent the mean of five consecutive cardiac cycles. After the echocardiography, a high-fidelity pressure transducing catheter was inserted via the right carotid artery into the left ventricle to measure the left ventricular pressure (LVP). When the rats returned to stable conditions, left ventricular systolic pressure (LVSP), left ventricular end-diastolic pressure (LVEDP), and their first derivative with respect to time (±  *dp*/*dt*  max) were continuously measured as before [23].

### 2.12. Evaluation of Infarct Size by Pathological Staining

Rats were immediately sacrificed after both echocardiography and hemodynamic measurements were obtained. The hearts were arrested and tissue sections of the myocardium were stained with hematoxylin-eosin (H&E) and Masson's Trichrome; light microscopy was used to evaluate the morphological changes at a magnification of 400x. Interstitial collagen deposition was visualized using Masson staining based on the percentage of blue staining and analyzed by the software of Image Pro Plus 6.0.

### 2.13. Evaluation of Apoptosis Rate by TUNEL Staining

The paraffin-embedded tissue was cut into sections, 4-5 *μ*m thick, and the terminal deoxynucleotidyl transferased UTP nick-end labeling (TUNEL) assays were performed to analyze myocardial apoptosis according to the manufacturer's instructions (Roche (Mannheim, Germany)). The apoptotic cells were analyzed by fluoresce microscopy. Green fluorescence represents the TUNEL-positive cells and blue fluorescence represents the nuclei. The apoptotic index was calculated as the ratio of the number of TUNEL-positive cardiomyocytes to the total number of nuclei.

### 2.14. Assessment of Relative Proteins by Western Blotting

Proteins of 6×His, HIF-1*α*, VEGF, p-Akt, Akt, ADM, Bcl-2, and Bax were detected at 4 weeks after myocardial infarction; moreover, proteins of HIF-1*α* and VEGF were also detected at 1 week after infarction. Left ventricular myocardial tissues were collected and lysed with lysis buffer. After sonication, the lysates were centrifuged, and the proteins were separated using SDS-PAGE and then transferred to Immobilon-NC membranes (Millipore, Boston, MA, USA). After being blocked with 5% skim milk in Tris-buffered saline at room temperature for 2 h, the membrane was incubated with primary antibodies against 6×His, HIF-1*α*, VEGF, p-Akt, Akt, ADM, Bcl-2, Bax, and GAPDH (1 : 1000) overnight at 4°C. (Antibodies against 6×His, HIF-1*α*, VEGF, Akt, phospho-Akt (Ser473), Bcl-2, Bax, and GAPDH were purchased from Cell Signaling Technology (Beverly, MA, USA)); antibody against ADM was purchased from Abcam (Cambridge, Massachusetts, UK). The membranes were incubated with secondary antibodies that conjugated with horseradish peroxidase for 1 h at 37°C (The rabbit anti-goat, goat anti-rabbit, and goat anti-mouse secondary antibodies were purchased from Beyotime (Shanghai, China)). The blots were imaged using a Bio-Rad imaging system (Bio-Rad, Hercules, CA, USA) and quantified using the Quantity One software package (West Berkeley, CA, USA). The value for the sham group was defined as 100%.

### 2.15. Statistical Analysis

SPSS 18.0 was used to analyze the data which are presented as the mean ± standard error of the mean (SEM) in this study. Comparisons among multiple groups were assessed by one-way analysis of variance. The LSD *t*-test was used to make intergroup comparisons. A value of *P* < 0.05 was considered statistically significant.

## 3. Results

### 3.1. Transfection Efficiency of PR39-ADM in CRL-1730 Cells

**Figure 1:**
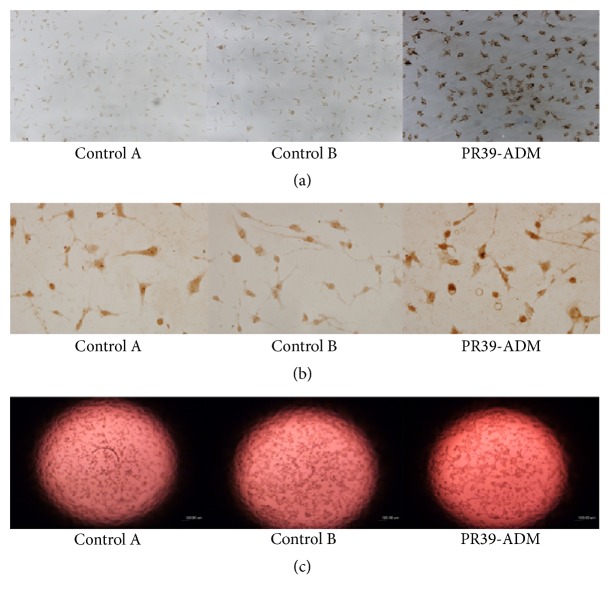
Expression of protein 6×His (a) and HIF-1*α* (b) in CRL-1730 cells. Matrigel assays. (c) Cells were seeded on growth factor depleted Matrigel in the absence of serum and in the presence of PR39-ADM, NS, or AAV-null. There was more cord formation in PR39-ADM treated cells than in other control cells (original magnification, ×40) (control A: Ns group; control B: AAV-null group).

Immunostaining showed that the CRL-1730 cells transfected with PR39-ADM significantly expressed 6×His (indirectly representing the expression of PR39 and ADM, Figure 1(a)) and HIF-1*α* (Figure 1(b)), which was not observed in other groups.

### 3.2. Effects of PR39-ADM on CRL-1730 Cells

**Figure 2:**
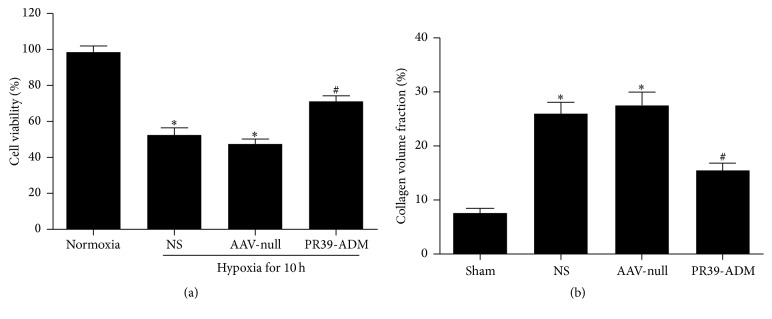
The change of cell viability in hypoxia condition (a). Collagen volume fraction assessment (b). In the collagen volume fraction assessment, data were expressed as mean ± SEM (*n* = 8 for each group). ^**∗**^*P* < 0.05 versus Normoxia group; ^#^*P* < 0.05 versus NS group.

After transfection and hypoxia induction, cell viability and proliferation were determined in each group. Viability and proliferation rates in the PR39-ADM group were higher than those in the other groups (*P* < 0.05). However, there was no significant difference between the AAV-null group and the NS group (*P* > 0.05) (Figure 2).

**Figure 3:**
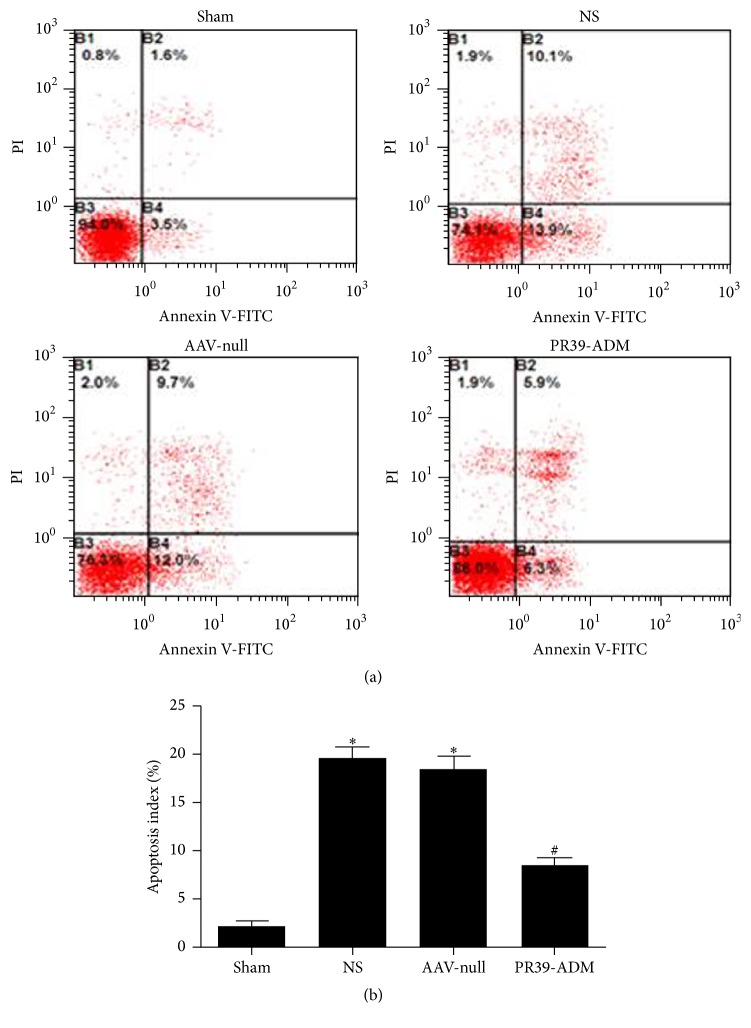
Apoptosis detection through flow cytometry. (a) Cells pretreated with corresponding treatments were collected, suspended, and then stained by PI and Annexin-V and finally analyzed by FCM. The experiments were carried out in triplicate. (b) The apoptosis index in different groups. ^**∗**^*P* < 0.05 versus sham group; ^#^*P* < 0.05 versus NS group.

The rate of apoptosis, detected by flow cytometry, in the PR39-ADM group was significantly lower compared to that in other groups (*P* < 0.05), and no significant difference was detected between the AAV-null group and NS group (*P* > 0.05) (Figure 3).

To determine whether PR39-ADM treatment could trigger tubulogenesis, we performed an in vitro Matrigel assay with CRL-1730 cells in the presence of PR39-ADM, AAV-null, and normal saline to analyze the extent of tube formation. We observed a significant increase in the formation of capillary-like structures in the endothelial cells exposed to PR39-ADM as compared to that observed with other conditions. Quantification of the tubes using a microscope showed more branch points in PR39-ADM-treated cells than in cells subjected to other treatments (Figure 1(c)).

### 3.3. PR39-ADM Increased Cardiac Function after Infarction

**Figure 4:**
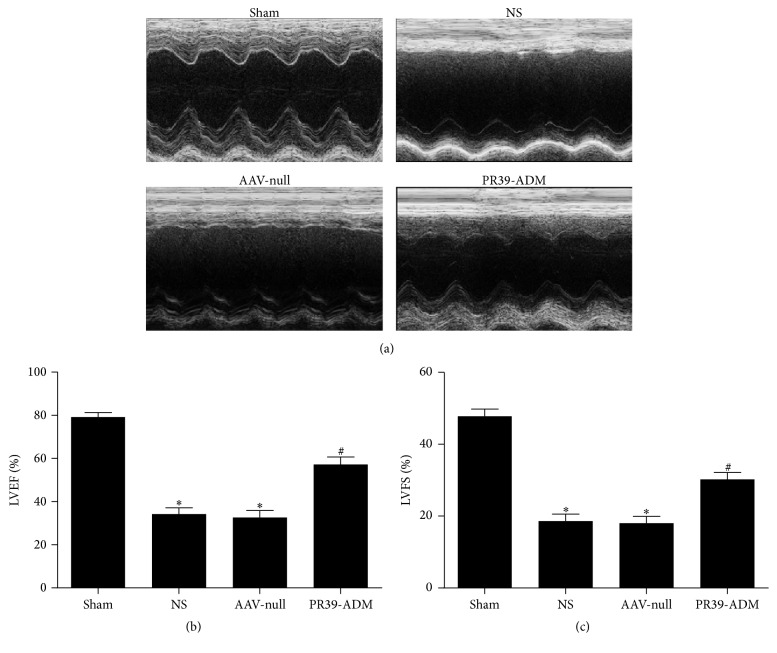
Echocardiography assessment. (a) The evaluation of cardiac function by echocardiography and representative M-mode images were shown. (b) Left ventricle ejection fraction (EF). (c) Left ventricular fractional shortening (FS). The results were expressed as the mean ± SEM (*n* = 8 for each group). ^*∗*^*P* < 0.05 versus sham group; ^#^*P* < 0.05 versus NS group.

Four weeks after myocardial infarction, cardiac function was assessed by echocardiography. As shown in Figure 4, PR39-ADM significantly restored myocardial impairment induced by LAD ligation in the PR39-ADM group, when compared to that in the other groups (*P* < 0.05).

**Figure 5:**
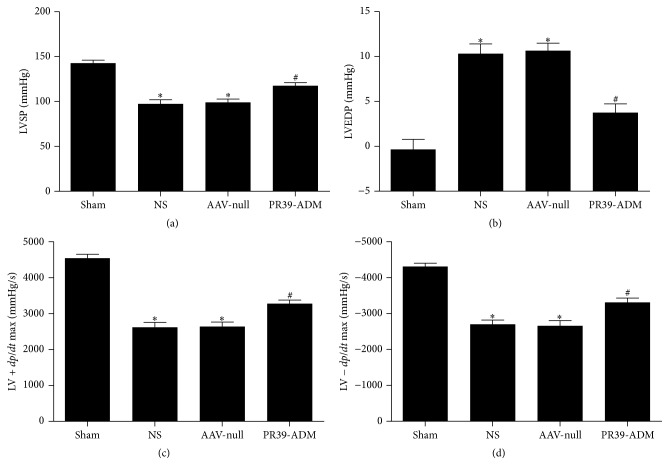
Hemodynamic assessment. The results were expressed as the mean ± SEM (*n* = 8 for each group). LVSP: left ventricular systolic pressure; LVEDP: left ventricular end-diastolic pressure; LV ± *dP*/*dt*  max: the instantaneous first derivation of left ventricle pressure. ^*∗*^*P* < 0.05 versus sham group; ^#^*P* < 0.05 versus NS group.

In addition, invasive hemodynamic assessment was performed immediately after echocardiography to assess cardiac function after infarction. As shown in Figure 5, PR39-ADM significantly increased LVSP and LV ± *dP*/*dt*  max and dramatically decreased LVEDP (compared to that in the other MI groups, *P* < 0.05).

### 3.4. PR39-ADM Attenuated Myocardial Injury Indicated by Pathological Staining

**Figure 6:**
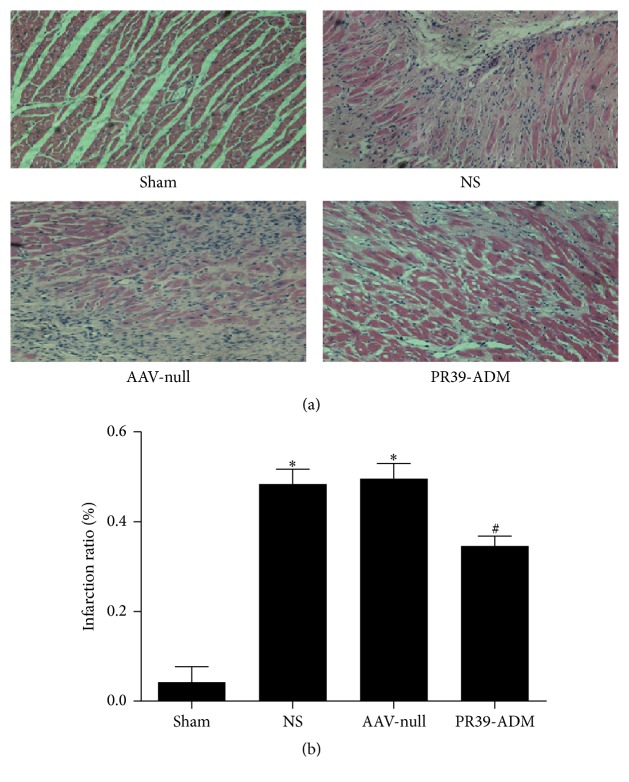
Hematoxylin-eosin staining suggests that PR39-ADM attenuates myocardial injury. (a) Representative images of H&E staining are shown (magnification ×200, *n* = 8 for each group). (b) Myocardial infarction size comparison. Data are expressed as mean ± SEM (*n* = 8 for each group). ^*∗*^*P* < 0.05 versus sham group; ^#^*P* < 0.05 versus NS group.

Myocardial damage was evaluated by H&E and Masson staining. Cardiomyocytes were intact and there was no necrosis or inflammatory cell infiltration in the sham group, and the cardiac muscle cross striations were clearly visible. However, in the MI groups, eosinophilic staining, neutrophil infiltration, and granulation tissue formation were commonly seen in myocardial infarcted areas. In the PR39-ADM group, the degree of neutrophil infiltration and myocardial lesion area were lower compared to those in the AAV-null (*P* < 0.05) and NS groups (*P* < 0.05). Myocardial infarct size was also attenuated with the use of PR39-ADM compared to that in other MI groups (*P* < 0.05) (Figures 6(a) and 6(b)).

As shown in Figure 2(b), PR39-ADM treatment resulted in the relatively regular arrangement of collagen fibers and decreased collagen volume fraction compared to those in the AAV-null group (*P* < 0.05) and NS group (*P* < 0.05).

### 3.5. PR39-ADM Alleviated Myocardial Apoptosis in MI Rats

**Figure 7:**
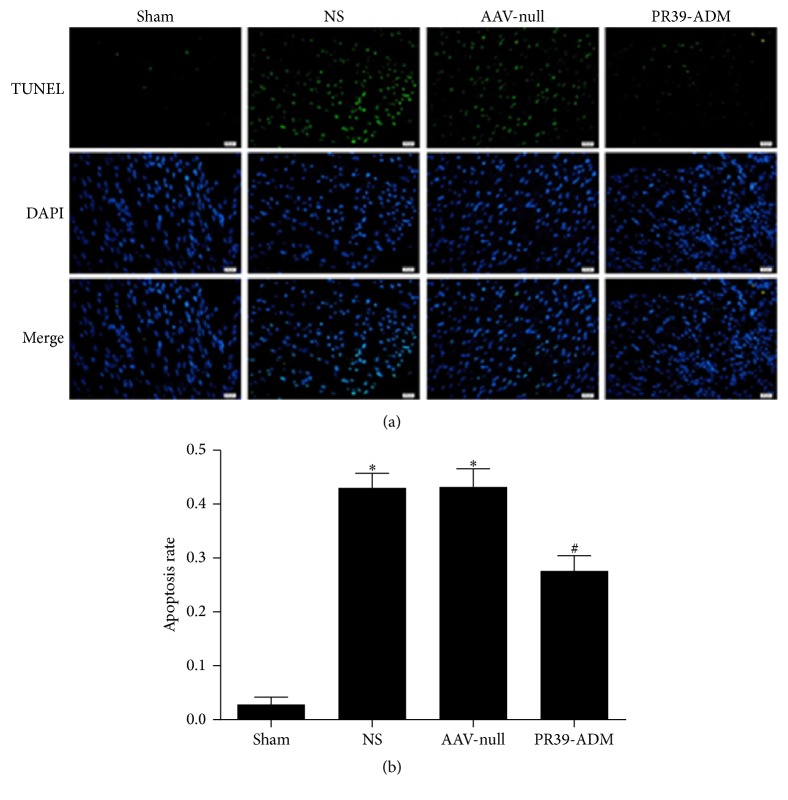
Evaluation of apoptosis rate by TUNEL staining. (a) Representative images of apoptosis are shown. The apoptotic cells were detected by TUNEL (green), and the nuclei were detected by DAPI (blue). The scale bar was 20 *μ*m. (b) The results were expressed as the mean ± SEM (*n* = 8 for each group). ^*∗*^*P* < 0.05 versus sham group; ^#^*P* < 0.05 versus NS group.

Myocardial apoptosis was evaluated by TUNEL. As shown in Figure 7, the apoptotic index was significantly increased in MI groups, and PR39-ADM significantly decreased this parameter compared to that in the AAV-null group (*P* < 0.05) and NS group (*P* < 0.05).

### 3.6. Relative Expression of Proteins Assessed by Western Blot

**Figure 8:**
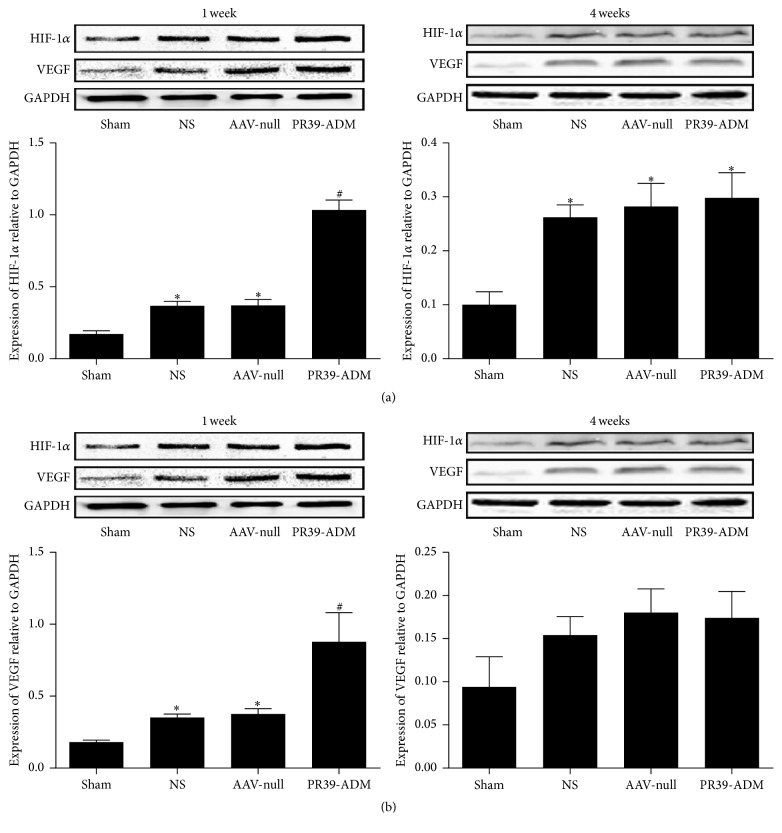
Effect of PR39-ADM on the expression of HIF-1*α* and VEGF at 1 week and 4 weeks after myocardial infarction. The values were expressed as the mean ± SEM (*n* = 6 for each group at 1 week, *n* = 8 for each group at 4 weeks). ^*∗*^*P* < 0.05 versus sham group; ^#^*P* < 0.05 versus NS group.

**Figure 9:**
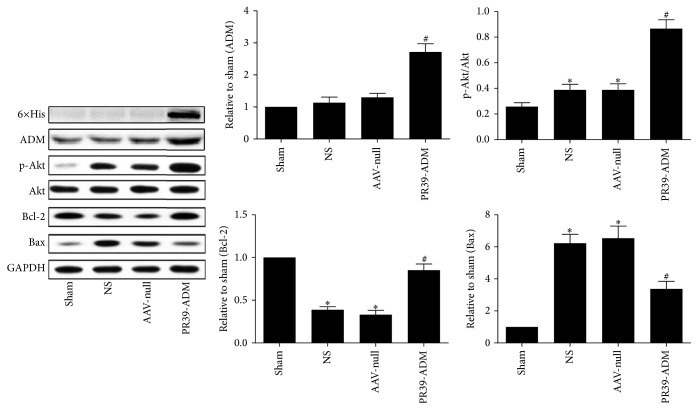
Effect of PR39-ADM on the expression of 6×His, ADM, phosphorylation-Akt, Bcl-2, and Bax following myocardial infarction. The values were expressed as the mean ± SEM (*n* = 8 for each group). ^*∗*^*P* < 0.05 versus sham group; ^#^*P* < 0.05 versus NS group.

To further investigate the molecular mechanism underlying cardioprotection mediated by PR39-ADM against MI, 6×His, HIF-1*α*, VEGF, p-Akt, Akt, ADM, Bcl-2, and Bax protein levels were detected by western blot. As shown in Figure 9, p-Akt was significantly increased after MI compared to that in the sham group, and PR39-ADM significantly increased p-Akt levels compared to that in the AAV-null (*P* < 0.05) and NS groups (*P* < 0.05); however, there was no significant difference between AAV-null and NS groups. The 6×His tag, used to label PR39-ADM, was assessed to indirectly validate the successful transfection of PR39-ADM. As expected, the expression of 6×His was significantly higher than that in the other groups, and so does the expression of ADM, which significantly increased in the PR39-ADM group. We detected the expression of HIF-1*α* and VEGF at both 1 and 4 weeks after infarction, shown in Figure 8. HIF-1*α* was significantly increased after MI, and PR39-ADM dramatically increased the expression of HIF-1*α* compared to that in other groups at 1 week after infarction; no significance was detected among MI groups at 4 weeks. VEGF is a downstream effector of HIF-1*α*; consistent with our expectation, MI significantly increased the expression of VEGF in the PR39-ADM group, and the expression was even higher compared to that in the other groups at 1 week after infarction. No significance was detected at 4 weeks among the groups. MI surgery induced a dramatic increase in Bax and a dramatic decrease in Bcl-2 expression relative to those in the sham group; PR39-ADM administration significantly decreased Bax and increased Bcl-2 expression.

## 4. Discussion

MI or acute myocardial infarction (AMI) is usually caused by coronary artery occlusion and leads to persistent ischemia and consequently results in local necrosis of the heart and corresponding cardiac dysfunction, being responsible for nearly 7.3 million deaths each year worldwide. Current therapies include surgical procedures such as coronary bypass, balloon angioplasty, stents, and heart transplant as the last option [24]. Pharmacological treatments generally facilitate surgical interventions to improve outcomes in patients. Limiting myocardial damage and adverse remodelling with novel therapies in the acute phase of MI continues to be an important objective.

Recently, new molecular and cellular targets together with genomic, proteomic, and other biotechnological advances have led to the discovery of novel pharmaceutical agents for the treatment of MI [25]. Chang et al. reported that the functional benefits of cell therapy were accompanied by differential regulation of protein expression in the recipient myocardium, which might contribute to the improved cardiac function [26]. The administration of growth factors, with the aim of promoting angiogenesis, chemotaxis, stem cell differentiation, cardiomyocyte survival and proliferation, reduction of apoptosis, and remodelling, holds great promise as a therapy for MI [25]. However, limitations such as the low half-life and systemic side effects should be seriously considered [27]. Therefore, specified gene delivery with the utilization of vectors such as adenovirus, lentivirus, and adeno-associated virus has been extensively used for the treatment of cardiovascular diseases, and this approach was estimated to represent 8.4% of all gene therapy trials reported in 2012 [8].

AAV, a small and nonpathogenic human virus that belongs to the parvovirus family, was originally discovered in the mid-1960s as a contaminant of cell culture also infected with adenovirus [28, 29]. The high efficiency of in vivo transduction of postmitotic tissues such as heart, brain, and retina, combined with its low immunogenicity, led to the widespread use of AAV as a transfer method for gene therapy in these organs [30]. In this study, PR39 and ADM were integrated into the recombinant AAV vector to express these two proteins in the ischemic region after MI.

AAV vectors that coexpress PR39 and ADM can be stably transfected into the ischemic area by means of local myocardial injection. With its characteristic of long-term persistence in muscle cells, the expression of PR39 and ADM was consistently increased with their corresponding protective functions. PR39 increased the expression of not only HIF-1*α*-dependent genes including VEGF and its receptor VEGF-R1, but also HIF-1*α*-independent genes such as VEGF-R2, FGFR-1, and syndecan-4 [11]. PR39 has been reported to function in angiogenesis, mainly by enhancing HIF-1*α*-dependent gene expression by selectively inhibiting proteasomal degradation of this transcription factor, which was shown in a pig model of chronic myocardial ischemia [31]. Consistent with a previous study, the quantity of HIF-1*α* as well as corresponding VEGF in the ischemic region was significantly increased with the use of PR39-ADM, and Matrigel assays indicated that PR39-ADM significantly accelerated the formation of visible rings and cords of CRL-1730 cells. Wu et al. reported that PR39 decreases caspase-3 activity and inhibits hypoxia-induced apoptosis in endothelial cells through an increase of IAP-2 expression; they also demonstrated PR39 to be an antiapoptotic factor in endothelial cells during hypoxia [12]. In our study, the rate of apoptosis was detected in CRL-1730 cells, and TUNEL assays were performed in addition to probing for apoptosis-associated proteins such as Bcl-2 and Bax; results suggested that PR39-ADM treatment significantly decreased the apoptosis induced by MI.

ADM executes numerous actions including vasodilation, natriuresis, and evasion of apoptosis and stimulation of nitric oxide (NO) production, mainly through the calcitonin receptor-like receptor (CLR) and a specific receptor-activity modifying protein, and activates the second messenger signal, resulting in an increase in cAMP and NO synthesis. ADM also acts on various intracellular signal transduction pathways such as protein kinase B phosphorylation and protein tyrosine kinase activation [32, 33]. Among them, PI3K and its downstream serine/threonine kinase Akt (also known as protein kinase B or PKB) regulate cellular activation, inflammatory responses, chemotaxis, and apoptosis [23, 34]. It has been demonstrated that PI3K/Akt pathway activation is protective against myocardial ischemia-reperfusion injury [35–38]. The results of our study showed that PR39-ADM treatment significantly increased Akt phosphorylation, which indicated that the PI3K/Akt signalling pathway is involved in the protective effect of PR39-ADM and should be further investigated using specific inhibitors of this signalling pathway.

Caution should be taken when interpreting the results of this study. We separately detected the two key proteins (HIF-1*α* and VEGF) regulated during cardiac protection 1 week and 4 weeks after the injection of PR39-ADM. Interestingly, at 1 week after injection, HIF-1*α* significantly increased in the MI groups, and in the PR39-ADM group, its expression increased to a higher level compared to that in other groups. HIF-1*α* was induced by hypoxia and PR39-ADM significantly increased its expression by selectively inhibiting proteasome degradation of this transcription factor. Consequently, VEGF, the key downstream growth factor, was significantly increased, which resulted in angiogenesis. However, at 4 weeks after treatment, the quantity of HIF-1*α* and VEGF of MI groups was slightly higher than that in the sham group, but no significant difference was detected among MI groups. Potential reasons are as follows: at the later stage of ischemia, PR39-ADM increased angiogenesis, dilated periphery microvessels, was antiapoptotic, restored the myocardium, and recovered blood flow to the ischemic area. Consequently, hypoxia was to some extent reversed, HIF-1*α* degradation was increased, and the quantity of HIF-1*α* and downstream VEGF returned to relatively low levels [39]. Moreover, in the results comparison part, no significant difference was found between the NS group and AAV-null group, indicating that the introduction of AAV as a gene-carrier has no significant side effects in cardiomyocytes. It should also be noted that AAV-associated gene therapy has the potential advantage of maintaining the expression of target genes at a sufficient concentration over a long period from a single administration. However, long-term expression of transfected genes, which may lead to unwanted side effects, was determined to be the potential shortfall of this kind of gene therapy. For example, VEGF has been one of the most popular downstream and direct angiogenesis stimulators investigated for gene delivery; however, persistent expression of VEGF can promote the formation of endothelial cell-derived intramural vascular tumours near the implantation site as well as dysmetabolism [40, 41]. Therefore, precursors of proangiogenic factors such as PR39 have been extensively researched recently; however, limitations described above still remained. Consequently, the shutdown theory was introduced and the HRE promoter was designed in our AAV vectors to tackle this problem. With the recovery of peripheral vessels as well as the influx of oxygen during the later stages of treatment, hypoxia triggered HRE activation was shut down and consequently terminated the persistent expression of PR39 and ADM along with HIF-1*α* and VEGF; thus, possible side effects were reduced.

Apoptosis and peripheral vessel blockage are two primary causes of MI occurrence and progression. Therefore, gene transfer using combinations of antiapoptotic and angiogenic genes could be the basis for new strategies to mitigate MI progression. In this study, an AAV vector coexpressing PR39 and ADM attenuated myocardial dysfunction induced by MI both in vivo and in vitro. In conclusion, hypoxia-induced, HRE-mediated PR39-ADM expression could quickly dilate blood vessels, increase blood supply, and promote myocardium repair in the early stages of MI and consistently protected myocardium through its antiapoptotic effect and by promoting new vascular formation. It then restored cardiac function as well as reduced myocardial remodelling at the later stage. Subsequently, HRE was repressed with the recovery of oxygen and consequently prohibited the overexpression of both upstream and downstream factors in addition to mitigating corresponding side effects. This strategy provides an alternative gene therapy method for MI and might have important practical clinical implications in the future.
